# Ancient Hybridization and Adaptive Introgression of an Invadolysin Gene in Schistosome Parasites

**DOI:** 10.1093/molbev/msz154

**Published:** 2019-06-27

**Authors:** Roy N Platt, Marina McDew-White, Winka Le Clec’h, Frédéric D Chevalier, Fiona Allan, Aidan M Emery, Amadou Garba, Amina A Hamidou, Shaali M Ame, Joanne P Webster, David Rollinson, Bonnie L Webster, Timothy J C Anderson

**Affiliations:** 1 Disease Intervention and Prevention, Texas Biomedical Research Institute, San Antonio, TX; 2 Department of Life Sciences, The Natural History Museum, London, United Kingdom; 3 London Centre for Neglected Tropical Disease Research (LCNTDR), Imperial College London, St Mary’s Campus, London, United Kingdom; 4 Réseau International Schistosomoses, Environnement, Aménagement et Lutte (RISEAL-Niger), Niamey, Niger; 5 Public Health Laboratory - Ivo de Carneri, Pemba, United Republic of Tanzania; 6 Centre for Emerging, Endemic and Exotic Diseases, Department of Pathobiology and Population Sciences, Royal Veterinary College, University of London, London, United Kingdom

**Keywords:** *Schistosoma haematobium*, *Schistosoma bovis*, parasite, adaptation, M8 metalloprotease

## Abstract

Introgression among parasite species has the potential to transfer traits of biomedical importance across species boundaries. The parasitic blood fluke *Schistosoma haematobium* causes urogenital schistosomiasis in humans across sub-Saharan Africa. Hybridization with other schistosome species is assumed to occur commonly, because genetic crosses between *S. haematobium* and livestock schistosomes, including *S. bovis*, can be staged in the laboratory, and sequencing of mtDNA and rDNA amplified from microscopic miracidia larvae frequently reveals markers from different species. However, the frequency, direction, age, and genomic consequences of hybridization are unknown. We hatched miracidia from eggs and sequenced the exomes from 96 individual *S. haematobium* miracidia from infected patients from Niger and the Zanzibar archipelago. These data revealed no evidence for contemporary hybridization between *S. bovis* and *S. haematobium* in our samples. However, all Nigerien *S*. *haematobium* genomes sampled show hybrid ancestry, with 3.3–8.2% of their nuclear genomes derived from *S. bovis*, providing evidence of an ancient introgression event that occurred at least 108–613 generations ago. Some *S. bovis*-derived alleles have spread to high frequency or reached fixation and show strong signatures of directional selection; the strongest signal spans a single gene in the invadolysin gene family (Chr. 4). Our results suggest that *S. bovis*/*S. haematobium* hybridization occurs rarely but demonstrate profound consequences of ancient introgression from a livestock parasite into the genome of *S. haematobium*, the most prevalent schistosome species infecting humans.

## Introduction

Introgressive hybridization occurs when hybrid offspring repeatedly backcross with one or both parental types, acting as a conduit for genetic exchange between species. Once present in the “new” genetic background, introgressed loci are broken up through recombination and exposed to selection. Deleterious alleles are purged while advantageous alleles may increase in frequency if they escape loss by drift. For example, genes impacting skin pigmentation (Vernot and Akey 2014) and immune response (Gittelman et al. 2016) were transferred between Neanderthals and humans during the out-of-Africa migration(s). The gene underlying seasonal coat color changes in snowshoe hares are introgressed from jackrabbits (Jones et al. 2018). Resistance to warfarin-containing-pesticides was transferred to mice from closely related wild species (Song et al. 2011), whereas resistance to insecticide-treated bed nets was transferred between *Anopheles gambiae* into *A. coluzzii* (Norris et al. 2015). Introgression allows for complex phenotypic traits to be quickly introduced into a population over the course of a few generations allowing for rapid adaptation to new environments that may not be possible when relying on mutation or standing variation alone (Hedrick 2013).

Gene transfer occurs frequently between bacterial species through plasmid transfer or scavenging of environmental DNA, with profound ecological and biomedical consequences. Among parasitic organisms, hybridization and introgression is a concern since this provides a potential path for genes encoding biomedically important traits including virulence, host specificity or drug resistance to be transferred between species (King et al. 2015). Schistosomes are dioecious trematodes that cause the debilitating disease schistosomiasis which infects more than 220 million people in 78 developing countries (World Health Organization 2019). *Schistosoma haematobium*, the focus of this article, infects 112 million people (World Health Organization 2019) and is responsible for extensive morbidity including bladder cancer, genital schistosomiasis, and other pathologies associated with the urogenital tract. Schistosomiasis is associated with increased susceptibility to HIV and progression to AIDS (Colley et al. 2014). Although primarily confined to Africa and adjacent regions recent schistosomiasis outbreaks in Corsica caused by hybrids between *S. haematobium* and a closely related livestock species *S. bovis* have raised concern that this hybridization may have promoted range expansion and transmission potential (Boissier et al. 2016; Kincaid-Smith et al. 2019).

Interspecific laboratory crosses between members of the *S. haematobium* species group, which contains nine closely related species, have produced viable hybrid offspring (e.g., see Rollinson et al. 1990; Bremond et al. 1993; Tchuenté et al. 1997; Southgate et al. 1998; Webster et al. 2013) through to the F7 generation (Mutani et al. 1985). In some cases, laboratory-reared, hybrid offspring have displayed hybrid vigor in the form of increased egg production (Mutani et al. 1985), increased infectivity to intermediate and definitive hosts (Wright and Ross 1980), and expansion of suitable intermediate and definitive hosts (Huyse et al. 2013). In natural populations, hybridization is diagnosed via a combination of pathology, egg morphology, and genetic identification via mitochondrial and nuclear discordance from single gene sequencing (reviewed in Leger and Webster 2017). In West Africa, parasites carrying ITS-rDNA from *S. haematobium*, but mtDNA from *S. bovis* are frequently observed and occasional parasites with ITS-rDNA copies from both species have been documented (table 1). These results suggest hybridization, but the limited genotyping cannot fully distinguish whether this results from recent hybridization (F1 or F2 parasites), ancient hybridization and introgression, or incomplete sorting of ancestral lineages.

**Table 1.** Summary of Studies Examining Hybridization between *S. bovis* and *S. haematobium*.

Study	Molecular Markers	Country	Num. Samples	Hybrid Profile Types		
				Nonhybrid Origin	Mitonuclear Disordance	Heterozygous Nuclear loci
Huyse et al. (2009)	*cox*1/ITS-rDNA	Senegal	158	126	30	2
Webster et al. (2013) ^a^	*cox*1/ITS-rDNA	Senegal	681	598	35	48
Boissier et al. (2016)	*cox*1/ITS-rDNA	France (Corsica)	73	43	30	0^b^
Moné et al. (2015)	*cox*1/ITS-rDNA	France (Corsica)	4	0	3	1
Leger et al. (2016)	*cox*1/ITS-rDNA	Niger	42	24	13	5^c^
Soentjens et al. (2016)	*cox*1/ITS-rDNA	Mali	2	0	2	0
Boon et al. (2017)	*cox*1/ITS-rDNA	Senegal	66	47	19	0
Catalano et al. (2018)	*cox*1/ITS-rDNA	Senegal	6	5	1	0
Boon et al. (2018)	*cox*1	Senegal	1,132	907	225	NA
Boon et al. (2018)	ITS-rDNA	Senegal	193	190	NA	3
Kincaid-Smith et al. (2019)	Whole genome	France (Corsica)	1	0	1^d^	
Sene-Wade et al. (2018)	*cox*1/ITS-rDNA	Senegal	81	51	23	7
Djuikwo-Teukeng et al. (2019)	cox1/ITS-rDNA, microsats	Cameroon	218	218	0	0
Oey et al. (2019)	Whole genome	Egypt	1	0	0	1
Oey et al. (2019)	Exome enrichment^e^	Zanzibar	8	8	0	0

a Also identified 31 and 28 possible *S. haematobium* and *curassoni* hybrids from mitonuclear discordance and heterozygous ITS-rDNA markers, respectively.

b Includes one nonhybrid *S. bovis*.

c Five samples contained an *S. bovis cox*1 and a heterozygous *S. curassoni*/*haematobium* ITS-rDNA profile.

d Genome data were generated from pooled samples of lab-passaged parasites. The sample(s) contained the *S. bovis* mitochondrial genome and 77% and 23% of the nuclear genome reads derived from *S. haematobium* and *S. bovis*, respectively.

e Data from Le Clec’h et al. (2018).

Schistosomiasis ranks second only to malaria as a parasitic disease impacting human health. Yet unlike malaria for which thousands of parasite genomes have been sequenced (Pearson et al. 2016; Amato et al. 2018), population genomic analysis of schistosomes is in its infancy, mainly because adult worms live in the blood vessels of human hosts and cannot be easily sampled. In this study, we examine hybridization and introgression in *S. haematobium*, leveraging a method that allows whole-genome amplification followed by exome capture or whole-genome sequencing from single miracidia larvae (Le Clec’h et al. 2018). We sequenced exomes from parasite populations from both East Africa (Zanzibar) and West Africa (Niger). We used these data to examine the distribution of haplotype blocks containing *S. bovis* alleles within the *S. haematobium* autosomal genome to determine 1) whether hybridization is ancient or ongoing, 2) to evaluate evidence for introgression, and 3) to determine whether introgressed alleles show signatures of selection.

## Results

### Parasite Samples, Exome Capture, and Sequencing

**Fig. 1.**
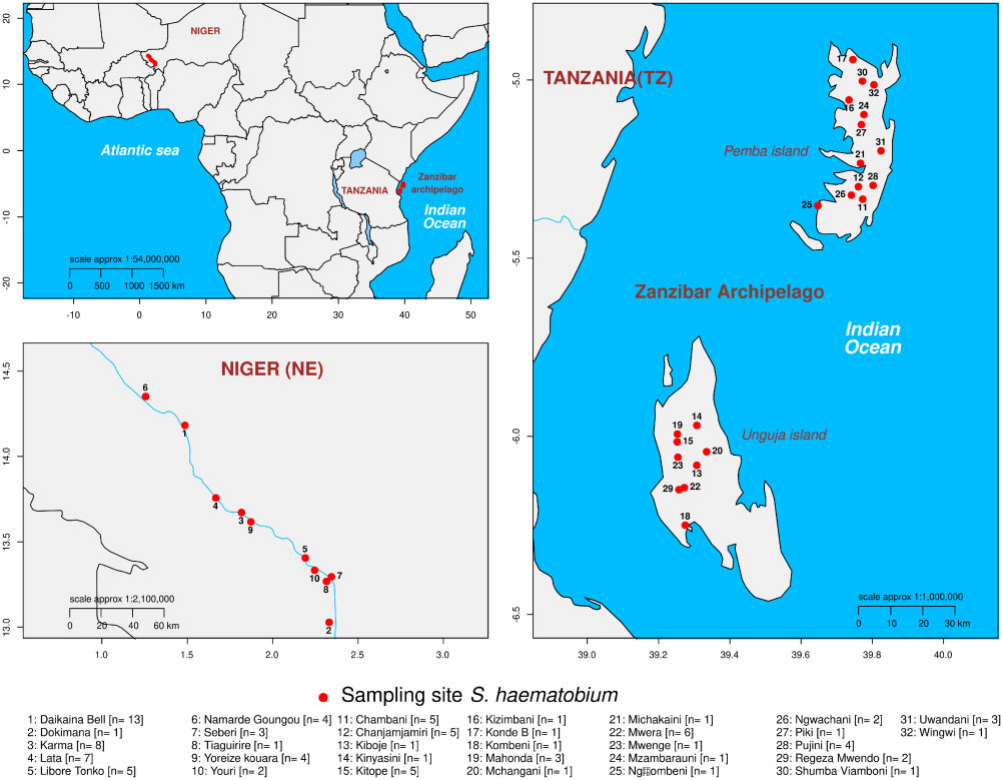
Collection localities. *Schistosoma haematobium* samples were collected in Niger and on the Zanzibar Archipelago off the coast of Tanzania.

We used individual, archived *S. haematobium* miracidia larvae from Niger and Zanzibar (Tanzania) fixed on Whatman FTA cards from the Schistosome Collection at the Natural History Museum (SCAN; Emery et al. 2012)*.* To minimize any impact of within host population structure (Steinauer et al. 2013), we used a single miracidium from each of 96 different *S. haematobium* infected people (*n*_Zanzibar_ = 48, *n*_Niger_ = 48) for this study (supplementary table S1, Supplementary Material online). The Zanzibar samples were collected in 2011 and came from 26 locations on Unguja and Pemba islands spaced up to 160.9 km apart (fig. 1). The Niger samples were collected in 2013 from school-aged children from ten locations along the Niger River located up to 125 km apart. We sequenced exomes from 88 miracidia from Niger (*n* = 48) and Zanzibar (*n* = 40) using whole-genome amplification and exome capture (Le Clec’h et al. 2018).

The exome capture probe set was designed using the *S. haematobium* reference sequence and contains 156,071 RNA baits targeting 94% of the exome length (15,002,706 bp/15,895,612 bp) in the nuclear genome as well as 67 RNA baits targeting the mitochondrial genome. We combined our data with eight exome sequences from Zanzibar that have been reported previously for a total of 47 from Zanzibar (Le Clec’h et al. 2018) and 48 from Niger. Probes were sequenced to an average depth of 40.75× (range: 5.58–98.27; supplementary table S1, Supplementary Material online) across the 15 Mb, targeted region after excluding a single outlier sample that did not generate much sequence data. Due to their overlapping nature, the 156,071 RNA probes targeted 52,242 regions of the genome. We looked to see if any of these regions were systematically underrepresented across all samples, potentially representing failure in the probe design. We found 51,873 of the 52,242 targeted sites were covered at ≥95% of bases in 48 or more samples, meaning that 99.3% of targeted regions were fully captured in the majority of samples.

### Mitochondrial Variation

**Fig. 2.**
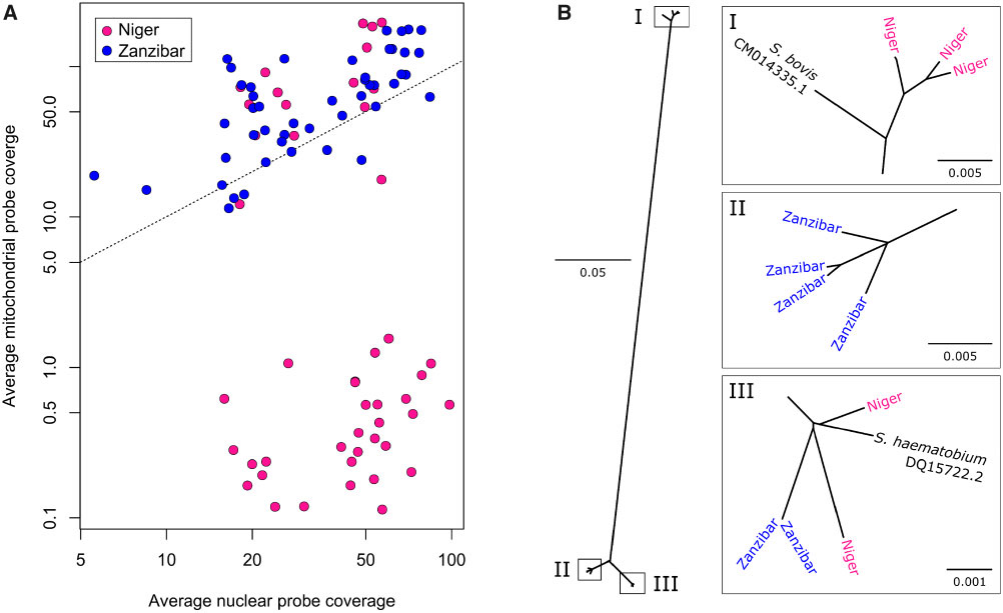
Mitochondrial sampling and phylogeny. (*A*) The mitochondrial genome was sequenced to high coverage despite large variations in the number of reads generated for most samples. For 31 Nigerien samples, mitochondrial reads were at very low frequency regardless of sequencing depth. This was attributed to the inefficient capture of the highly diverged mitochondria in these 31 Nigerien samples. (*B*) Maximum likelihood phylogeny of 12 mitochondrial genomes from *Schistosoma haematobium* and *S. bovis* and *haematobium* reference sequences. Three Nigerien *S. haematobium* samples are related to *S. bovis* indicating mitochondrial introgression from *S. bovis* into the Nigerien *S. haematobium* population.

Our initial attempts to genotype mitochondrial loci contained within the exome probe set failed in 31 of the Nigerien *S. haematobium* samples due to poor representation of mitochondrial reads in the sequence data, despite high rates of capture from nuclear loci (fig. 2). We therefore sequenced whole genomes from a subset (*n* = 12) of parasites to an average coverage of 17.8× and de novo assembled the mitochondrial genomes for each individual, while we genotyped *cox1* in other samples by Sanger sequencing. We found that the samples that failed mitochondrial probe capture contained a *S. bovis*-derived mitochondrial haplotype that was 15–18.05% divergent from that of *S. haematobium* and therefore poorly captured by our exome capture baits, designed from the *S. haematobium* reference sequence (Bi et al. 2012). In all, 65% (*n *=* *31) of the Nigerien *S. haematobium* miracidia examined had a *S. bovis* mtDNA, similar to other West African *S. haematobium* populations (Huyse et al. 2009; Webster et al. 2013). In contrast, *S. bovis* mtDNA was not present in the Zanzibari *S. haematobium* population.

### Exome Variation

The presence of *S. bovis* mtDNA in Nigerien *S. haematobium* could result from contemporary hybridization or past introgression events. To differentiate between these two scenarios, we examined autosomal, exonic single nucleotide polymorphisms (SNPs) recovered by the exome capture probes (Chevalier et al. 2014; Le Clec’h et al. 2018). In addition to our 96 *S. haematobium* samples, we used sequence data from previous studies (Young et al. 2012; Coghlan et al. 2019) of six closely related species in the *S. haematobium* species group (*S. bovis*, *S. curassoni*, *S. intercalatum*, *S. guineensis*, *S. mattheei*, and *S. margrebowiei*). The genome assembly of *S. haematobium* is highly fragmented in thousands of scaffolds compared with the *S. mansoni* assembly, which comprises large chromosomal length scaffolds. However, the genomes of *S. haematobium* and *S. mansoni* are ≥89.4% syntenic (Young et al. 2012). We aligned the *S. haematobium* and *S. mansoni* assemblies to convert SNP coordinates from their position on *S. haematobium* contigs to the corresponding position on *S. mansoni* chromosomes to take advantage of the more contiguous assembly and gene annotations available for *S. mansoni* (Berriman et al. 2009; Protasio et al. 2012). We verified local synteny of the transposed SNP coordinates by comparing linkage disequilibrium (LD) in 1-Mb windows between the two sets of SNP positions (supplementary fig. S1, Supplementary Material online). We recovered 370,770 autosomal, exonic SNPs for all samples including outgroups after genotyping, filtering and scaffolding along the *S. mansoni* assembly. These included 185,601 SNPs segregating in Niger and Zanzibar *S. haematobium* populations, of which 35,102 were common (minor allele frequency (MAF) > 0.05). We found higher SNP diversity (paired *t*-test; df = 20, 825; *P *=* *2.2 × 10^−16^) among common alleles in Niger (mean *π* = 7.97 × 10^−5^; interquartile range = 6.3 × 10^−5^) than in Zanzibar (mean *π* = 2.89 × 10^−5^; interquartile range 2.3 × 10^−5^).

### Quantifying and Dating Admixture

**Fig. 3.**
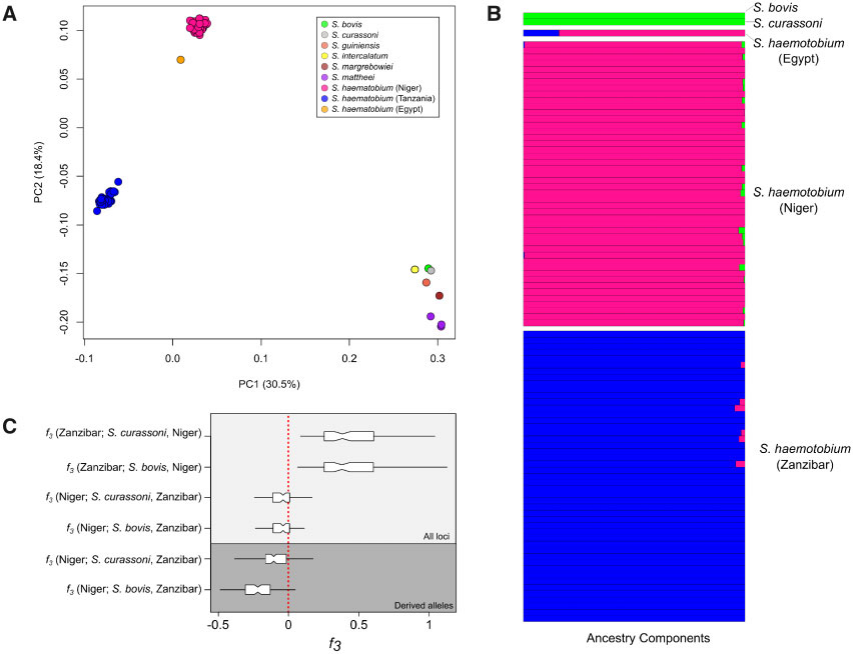
Population structure in *Schistosoma haematobium*. (*A*) PCA plot shows clear distinction between the two *S. haematobium* populations and the rest of the species examined: we see no evidence for recent hybridization. (*B*) Quantification of population structure with ADMIXTURE showed low levels of admixture between *S. haematobium* populations and *S. bovis*/*curassoni*. (*C*) The three-population statistic (*f*_3_) was used to formally test admixture between each of the *S*. *haematobium* populations, *S*. *bovis*, and *S*. *curassoni*. When testing *f*_3_ (test; *A*, *B*), a negative result indicates that the test group is an admixed population from *A* and *B*. To differentiate between introgression from *S*. *bovis* and *curassoni* in Nigerien *S*. *haematobium*, we recalculated *f*_3_ using only derived alleles from *S. bovis* or *curassoni*. These results rule out contemporary hybridization between populations of *S. haematobium* and *S*. *bovis* or *curassoni* and indicate that low levels of introgression are restricted to the Nigerien *S*. *haematobium* population.

We removed SNPs showing strong LD to generate a reduced SNP subset for investigating population structure. A principle component analysis (PCA) from 5,882 unlinked, autosomal SNPS MAF > 0.05) and all *Schistosoma* samples clearly differentiated *S. haematobium* from all other species along the first two PCs which accounted for 48.9% of genotypic variation observed (fig. 3*A*). The lack of intermediate genotypes between *S. haematobium* and other schistosome species suggests that we did not have early generation hybrids within our samples. We used ADMIXTURE (Alexander et al. 2009) to assign ancestry proportions to the *S. haematobium*, *bovis*, and *curassoni* samples. *Schistosoma curassoni* was included due to its close phylogenetic affiliation with *S. bovis* (Lockyer et al. 2003). Three distinct ancestry components were identified corresponding to *S. bovis*/*curassoni*, Nigerien, and Zanzibari *S. haematobium*. The *S. bovis*/*curassoni* ancestry component was present in 16 of the 48 Nigerien *S. haematobium* indicating low levels of potential admixture (0.1 < *Q *>* *2.7; fig. 3*B*), whereas no admixture was identified in the Zanzibari *S. haematobium*.

We used the three-population test (*F*_3_) to determine whether *S. bovis* and/or *S. curassoni* were sources of introgressed alleles in the Nigerien *S. haematobium* population (Reich et al. 2009). The *F*_3_ test determines whether a target population is admixed from two source populations by calculating the product of differences in allele frequencies between the target and each source population. A negative *F*_3_ indicates the target is an admixed population from the two sources. We used the Zanzibar *S. haematobium* population as a representative of the “source” *S. haematobium* population and alternated *S. bovis* and *S. curassoni* as the second source. *F*_3_ results when testing for admixture from *S. bovis* (*F*_3_ = −0.05, SE = 0.001, *Z* = −5.73) and *S. curassoni* (*F*_3_ = −0.05, SE = 0.01, *Z* = 5.28) were negative indicating admixture from both species in the Nigerien *S. haematobium* population (fig. 3*C*). *Schistosoma bovis* and *curassoni* are very closely related. The admixture signature from *F*_3_ could be driven by shared variation from the *S. bovis* and *curassoni* common ancestor. We recalculated *F*_3_ from, but only using *S. bovis* or *curassoni* derived alleles to minimize the signal from shared sites. These data provided evidence for admixture between *S. haematobium* and *S. bovis* (*F*_3_ = −0.23, SE = 0.0129, *Z* = −18.18) but failed to identify admixture from *S. curassoni* (*F*_3_ = −0.03, SE = 0.025, *Z* = 1.36). Incomplete lineage sorting provides a possible explanation when closely related alleles are found within different species. We confirmed that the *S. bovis* alleles in the Nigerien *S. haematobium* samples were the result of introgression as opposed to incomplete lineage sorting using Patterson’s *D* (*D* = 0.144; SE = 0.04; Patterson et al. 2012). From these results, we infer that *S. bovis* alleles in Nigerien *S. haematobium* are the result of admixture.

**Fig. 4.**
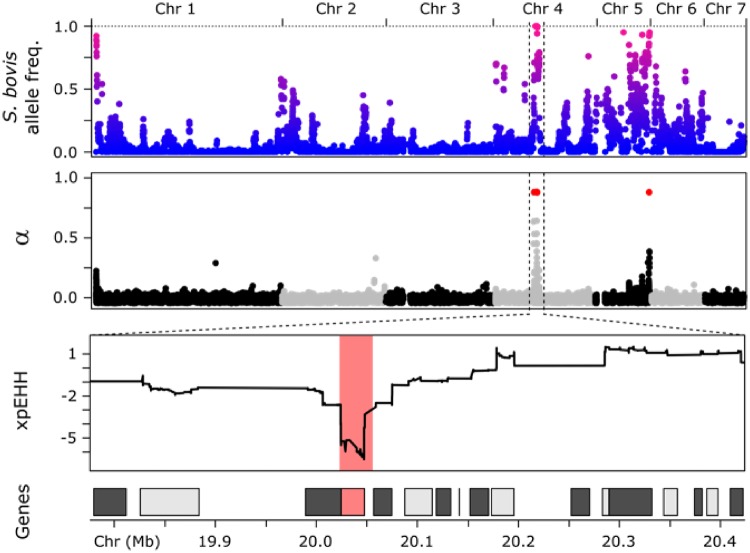
Selection on introgressed alleles in Nigerien *Schistosoma haematobium*. We aligned plots showing proportion of *S. bovis* ancestry, the *α* statistic from BayeScan, and xpEHH. *Schistosoma bovis* alleles have reached high frequencies in the Nigerien *S. haematobium* population. Introgressed alleles under directional selection were identified using allele frequency differences (*α*; alpha) between populations and using regions of extended homozygosity (xpEHH). Each test identified multiple regions under selection, but only one region was identified by both approaches. This region on Chr 4 spanned a single gene (Smp_120703, invadolysin) at which *S. bovis* alleles are approaching fixation in Nigerien *S*. *haematobium*.

We used PCAdmix (Brisbin et al. 2012) to identify locally introgressed alleles in the Nigerien *S*. *haematobium* population. Ancestry was assigned to phased haplotype blocks by comparing them to a reference panel of *S. bovis* and Zanzibari *S. haematobium*. Within each Nigerien *S. haematobium* miracidium 3.3–8.1% (*x¯* = 5.2%; fig. 4) of alleles were identified as *S. bovis* haplotypes. In contrast, we observed close to zero *S. bovis* ancestry in Zanzibari miracidia used as a control (0.004–0.2%). Combined, these results indicate at least one introgression event between Nigerien *S. haematobium* and *S. bovis.* In comparison, Zanzibari *S. haematobium* do not contain *S. bovis* alleles.

**Fig. 5.**
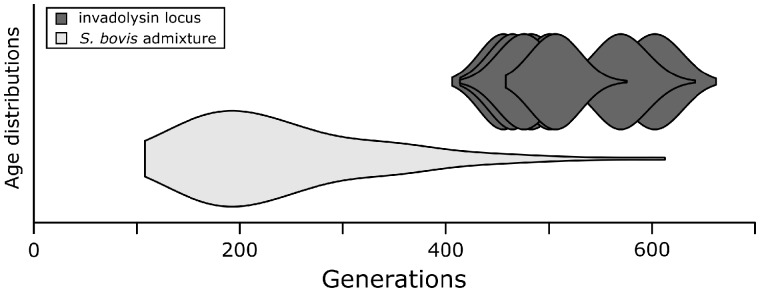
Time since admixture. Time since admixture was estimated using introgressed haplotype block length using methods described in Schumer et al. (2016). The age of the invadolysin (Smp_127030) locus specifically (*n*_runs_ = 10) was aged using startmrca (Smith et al. 2018). Age estimates for both methods overlap, but the distribution of time since introgression tends to be younger than the estimated age of the invadolysin (Smp_127030) locus. Estimates of time since introgression could be influenced by exome data, which is limited in its ability to identity small haplotype blocks and result in conservative, younger age estimates.

Recombination breaks down introgressed segments over time. As a result, the length of introgressed haplotype blocks can be used to date time since admixture in number of recombination events or generations (Schumer et al. 2016). Using this method we identified 5,649 *S. bovis* haplotype blocks in Nigerien *S. haematobium* averaging 0.5 cM in length and estimated admixture to have occurred ∼240.6 generations ago (min = 107.8, max = 612.5; fig. 5). Estimates of *Schistosoma* generation time vary greatly: while adult worms can live up to 37 years (Chabasse et al. 1985), the minimum time for eggs to mature into adult worms is estimated to be ∼85 days in a laboratory setting (Loker 1983; Crellen et al. 2016). Additional factors including water temperature (Lawson and Wilson 1980), host availability, and drug treatment will further influence average generation times. Assuming a ∼1-year generation time (King et al. 2000), the hybridization event leading to introgression occurred ∼240.6 years ago (min = 107.8, max = 612.5), however this date should be considered as a course estimate given the complexities in estimating *Schistosoma* generation time in natural populations. Importantly, although all 48 Nigerien miracidia contain introgressed *S. bovis* DNA resulting from ancient hybridization, we observed no F1 or early generation hybrids. Given 0/48 early generation hybrids detected in our population sample, we infer that the population frequency is between (0–7.4%; 95% binomial confidence interval; Clopper and Pearson 1934).

### Selection on Introgressed Alleles

Since the admixture event(s) some *S. bovis* alleles are approaching or have become fixed in the Nigerien *S. haematobium* population (fig. 4). We used two complementary methods to examine directional selection in the Zanzibari and Nigerien *S. haematobium* samples. First, BayeScan (Foll and Gaggiotti 2008) was used to quantify selection (*α*) as a function of allele frequency differences at individual SNPs. Then large haplotype blocks under directional selection were identified using cross-population extended haplotype homozygosity (xpEHH; supplementary fig. S2, Supplementary Material online; Sabeti et al. 2007). BayeScan and xpEHH identified 2 and 17 regions under directional selection between the Zanzibari and Nigerien *S. haematobium* populations. The strongest signals of selection from both analyses (*α* > 1.75; xpEHH ≤ −3) occurred at a 23-kb locus on chromosome 4 (Chr4: 20,023,951–20,047,325; fig. 4), which also overlapped the region with the highest frequency of introgressed *S. bovis* alleles in the Nigerien *S. haematobium* population. Other regions with high frequency of introgressed alleles, including regions on Chrs. 5 and 6 (fig. 4) did not clearly indicate directional selection in Bayescan and/or xpEHH analyses. Various factors including SNP density could have influenced these findings. Future work, using whole-genome sequencing, will focus on these regions of high introgressed allele frequency.

**Fig. 6.**
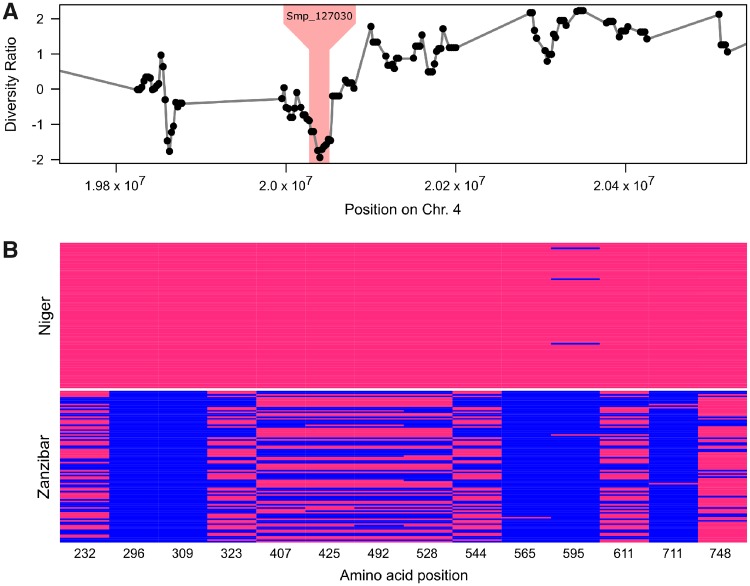
Reduced diversity at the invadolysin (Smp_127030) locus in the Nigerien *Schistosoma haematobium* population. (*A*) We compared differences in nucleotide diversity (*π*) across the Nigerien and Zanzibari *S*. *haematobium* populations calculated as $\log(\frac{\pi \mathrm{Niger}}{\pi \mathrm{Zanzibar}})$. The region containing invadolysin (shaded) shows reduced variation (in the bottom 0.2% genome wide) in the Nigerien *S. haematobium* population. (*B*) We identified 44 SNPs in Smp_127030, of which 14 resulted in nonsynonymous changes (shown) and were also shared by more than one individual.

Selection of introgressed *S. bovis* alleles would be expected to purge variation in the vicinity of these alleles (Smith and Haigh 1974; Stephan 2019). We therefore plotted the log ratio of diversity (*π*) in Niger and Zanzibar samples, to identify genome regions showing exceptionally low diversity in Niger. The 23-kb locus genome region on Chr. 4 falls within the bottom 0.2% of values genome wide, consistent with the expectations of a selective sweep (fig. 6).

To confirm that this locus was the product of an introgression event rather than the result of selection on standing variation, we calculated the time to the common ancestor of the 23-kb locus in the Nigerien haplotypes relative to the surrounding genomic regions using startmrca (Smith et al. 2018). Using mutation (8.1*e*^−9^/bp/generation) and recombination rates (244.2 kb/cM), reported for *S. mansoni* (Criscione et al. 2009; Crellen et al. 2016), we estimated the time since divergence of the 23-kb target region in the Nigerien haplotypes to be 476 generations ago (437.9–616.6; 95% CI; fig. 5), well within the range of our previous estimates of introgression from the haplotype block length analysis.

### Characteristics of the Introgressed Invadolysin Gene

**Fig. 7.**
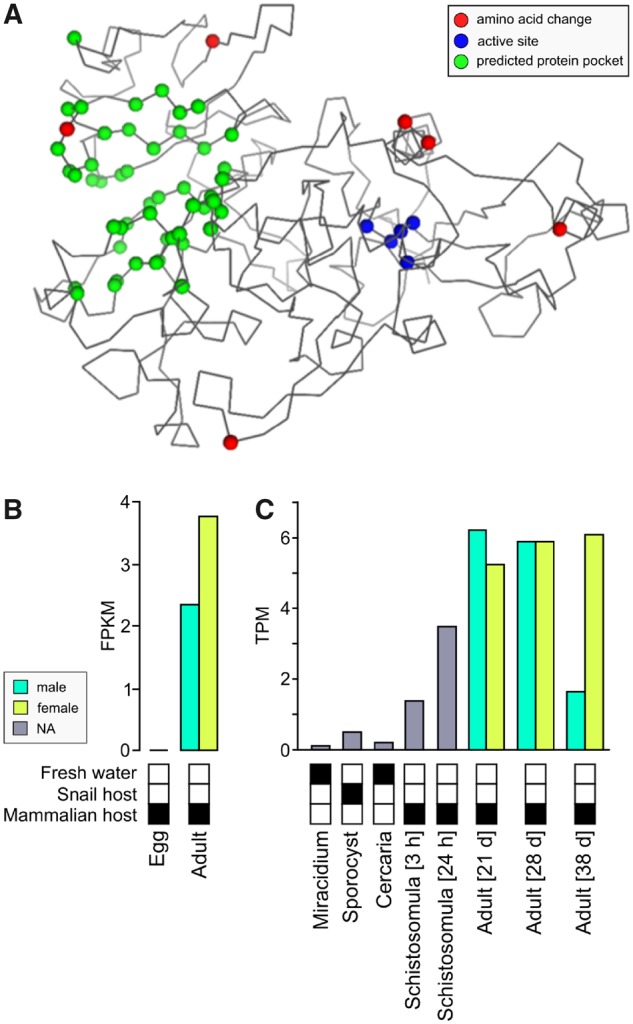
Invadolysin (Smp_127030) structure and expression. (*A*) The invadolysin (Smp_127030) structure was modeled based on a homologous *Drosophila* leishmanolysin structure. The amino acid changes are associated with the exterior of the protein and do not fall within the active site. (*B*) Invadolysin (Smp_127030) is expressed in adult worms in *Schistosoma haematobium*. (*C*) In *S. mansoni*, Smp_127030 is expressed in stages associated with the mammalian host, and most highly expressed in adult worms.

The 23-kb introgressed region spans a single gene alternatively referred to as Smp_127030 (*S. mansoni* protein ID), MS3_06416, (*S. haematobium* protein ID), leishmanolysin, or invadolysin. Since we are using *S. mansoni* genome annotations and chromosomal coordinates, we will refer to the invadolysin gene by its *S. mansoni* protein ID; Smp_127030. Within the invadolysin gene, we found 23 SNPs coding for 14 nonsynonymous changes (excluding singletons) two of which show fixed differences between Zanzibar and Niger populations (fig. 6). We were able to place six of the 14 nonsynonymous amino acid changes on a protein structure of Smp_127030, modeled from the *Drosophila* invadolysin (Protein DB: d1lmla). All were in peripheral positions on the protein (fig. 7) with little impact on the inferred protein structure (Kelley et al. 2015).

## Discussion

We were unable to find evidence of contemporary hybridization between *S. haematobium* and other schistosome species using samples from Zanzibar and Niger. Instead our data provide evidence for an ancient hybridization event between *S. haematobium* and *S. bovis* occurring hundred(s) of generations ago. Introgression was regional occurring in Niger but not in Zanzibar. In a Niger *S. haematobium* population, 3.3–8.2% of the genome are introgressed *S. bovis* alleles. Some of the introgressed *S. bovis* alleles have almost reached fixation within *S. haematobium* and show signatures of strong selection, providing unambiguous evidence for cross species transfer of genes through hybridization and introgression.

### Ancient Introgression Rather than Contemporary Hybridization

Multiple studies of West African *S. haematobium* miracidia have described discordant mitochondrial DNA and rDNA sequences in up to 41% of parasites sampled (table 1). These studies have been interpreted to indicate that hybridization occurs at high frequencies within many West African locations where *S. haematobium* and *S. bovis* occur sympatrically. In contrast, our exomic analyses using samples from Niger and Zanzibar reveals ancient hybridization and subsequent introgression.

Although 65% of miracidia sampled from Niger contained *S. bovis*-derived mtDNA, none of these 48 parasites showed evidence for recent hybridization based on analysis of the autosomal genome: we did not find any individual miracidia with 50% or 25% of their genome derived from *S. bovis* as would be expected for F1 or F2 hybrids. These results suggest that hybridization is either rare (0–7.4%; 95% binomial confidence interval; Clopper and Pearson 1934) in *S. haematobium*, or geographically localized. Broader geographical sampling and genomic characterization of miracidia will be needed to accurately determine the frequency of F1 or early generation hybrids in other *S. haematobium* populations.

Second, previous studies have suggested that 0–41% of parasites sampled in West African locations have hybrid origins based on mitochondrial and rDNA genotyping (table 1). This is clearly an underestimate: we found that 100% of *S. haematobium* miracidia sampled from Niger contain *S. bovis* ancestry, with 3–8% of autosomal loci derived from *S. bovis*. We conclude that ancient hybridization and introgression best explains the Nigerien genomic data and we predict that more extensive genomic surveys will demonstrate that recent (F1 or F2) hybrids between *S. haematobium* and *S. bovis* occur rarely in nature.

Crosses between multiple members of the *S. haematobium* species group, other than *S. bovis* and *S. haematobium*, have been staged in the laboratory using rodent hosts (Webster et al. 2006). Furthermore, field collected miracidia with mtDNA and rDNA markers from different species have been collected in nature. These include reports of nonviable, hybrid miracidia between *S. mansoni*, agent of intestinal schistosomiasis and *S. haematobium* (Webster et al. 1999). We emphasize that our conclusions regarding ancient introgression apply to *S. haematobium* and *S. bovis* only. Genomic analyses of putative hybrid miracidia from other schistosome species combinations or locations will determine if these reflect ancient or contemporary hybridization events and can help to understand how species barriers are maintained in schistosomes.

We estimate that the hybridization between *S. haematobium* and *S. bovis* occurred 240 (range: 108–613) generations ago, based on the size distribution of introgressed fragments. However, we emphasize that our estimate of time since introgression is likely to be conservative because exome data provides limited ability to identify small haplotype blocks. Despite these limitations the genomic data demonstrate that the introgression event occurred more than a hundred generations ago but subsequent to the divergence of the Nigerien and Zanzibari *S. haematobium* populations. Whole genome, rather than exome data, together with improvements in the *S. haematobium* genome assembly, and broader geographical sampling will improve our dating estimates of ancient hybridization with *S. bovis*.

Given that exome capture probes failed to capture *S. bovis* mtDNA, there is also a potential concern that our exome capture approach will preferentially capture *S. haematobium* DNA, and underestimate *S. bovis* alleles within the nuclear genome. We do not think this is the case for two reasons. First, *S. haematobium* and *S. bovis* genomes are ∼3% divergent (Oey et al. 2019). Exome capture effectively captures genomes at this level of divergence with minimal bias (Bi et al. 2012). Second, we examined proportions of *S. bovis* ancestry in 12 miracidia with both exome and genome sequence data (supplementary fig. S4, Supplementary Material online). Ancestry estimates were consistent between data types with the largest difference between estimates being 0.015 (1.5%). If exome capture was biased against detection of *S. bovis* admixture, then we would have expected to see much higher *S. bovis* ancestry estimates in the whole-genome data (e.g., 25%).

### Introgression is Geographically Widespread

How widespread is this ancient introgression event in Africa? Our results indicate regional introgression in parasites from Niger, but not from Zanzibar. Introgression may extend across multiple countries in Africa. The reference *S. haematobium* strain originated from Egypt (Young et al. 2012) and contains introgressed *S. bovis alleles*. Oey et al. (2019) sequenced the *S. bovis* genome and compared this with the Egyptian *S. haematobium* reference. They noted high similarity between *S. bovis* and the *S. haematobium* reference strain in genome regions spanning up to 100 kb and hypothesized that these regions were the result of an introgression event between *S. bovis* and *S. haematobium*. Interestingly, one of these high similarity segments is on chromosome 4 and spans the invadolysin locus (Smp_127030) indicting that the *S. bovis* invadolysin allele is not only present in Niger but also in Egypt. Taken together, our findings, in combination with Oey et al. (2019), indicate that the common ancestor of the Nigerien and Egyptian *S. haematobium* reference hybridized with *S. bovis*, suggesting that remnants of this introgression event may be widespread in extant *S. haematobium* in Africa. Interestingly, *S. bovis* has been recorded in Zanzibar (Pennance et al. 2018), but we see no evidence for introgression of *S. bovis* alleles in Zanzibari *S. haematobium*. Further, sampling will reveal the number and geographical extent of introgression events in *S. haematobium* across Africa.

### The Nature of Selection Driving Introgression

Invadolysin is a member of a M8 metalloprotease gene family originally identified in *Leishmania* (Russell and Wilhelm 1986). In *Leishmania*, these are surface proteins associated with degradation of collagen and fibrenonectin in the extracellular matrix and larval penetration of mammalian hosts (Yao et al. 2003). In schistosomes M8 metalloproteases have undergone a rapid gene family expansion (supplementary fig. S3, Supplementary Material online) and are among the most abundant transcripts and secreted proteins (Curwen et al. 2006; Liu et al. 2014; Coghlan et al. 2019) in larval stages. RNAi knockdowns of one invadolysin paralog in miracidia of *S. mansoni* led to reduced larval penetration and establishment in the snail intermediate host, reducing cercariae production (Hambrook et al. 2018).

Members of the invadolysin family are expressed in a stage specific manner, similar to globins in vertebrates (Storz et al. 2013). We suspect that the introgressed *S. bovis-*derived invadolysin is selected during the mammalian, rather than the snail infective stage of the lifecycle. The *S. mansoni* paralog of this gene (Smp_127030) is expressed during stages associated with the mammalian host and most highly expressed in adult worms (supplementary fig. S5, Supplementary Material online). Consistent with this, the Smp_127030 is expressed in adult worms in *S. haematobium* (fig. 7*B*), but expression data are not available for other life stages (except eggs). It is unclear how the *S. bovis* invadolysin (Smp_127030) allele benefits *S. haematobium* based on the currently available data.

We were able to confidently place SNPs in probed regions covering only half of the invadolysin (Smp_127030) exons due to ambiguity in lifting coordinates between the SchHae_1.0 and SMV7 *S. haematobium* and *S*. *mansoni* assemblies. Of those able to be transferred, fewer than half of the nonsynonymous SNPs we identified were able to be placed on the inferred protein structure. Despite these limitations, we find strong evidence of selection from the available population data. We speculate that the introgressed invadolysin is involved in tissue penetration (Wilson 2012) or immune evasion (Hambrook et al. 2018) within the mammalian host. Functional analysis of this system may be possible, since certain strains of *S. haematobium* can be maintained in the laboratory and will be a central priority for future work with this system.

### Implications for Schistosome Control

Significant resources are being directed toward schistosome control and elimination strategies (Savioli et al. 1997; Zhou et al. 2005; King 2009; Webster et al. 2014; Webster et al. 2016; Tchuenté et al. 2017). Hybridization between human and livestock schistosomes, including *S. bovis* and *curassoni*, will complicate these efforts (King et al. 2015). The introgression of the *S. bovis* invadolysin allele in *S. haematobium* provides an example of an adaptive introgression between an animal and human parasite species. Gene exchange between these two species highlights the need for effective coordination between medical and veterinary researchers and a “one health” approach to schistosome control and management (Webster et al. 2016).

## Conclusions

Hybridization between species is a powerful source of evolutionary novelty. In humans, up to 4% and 5% of the genome contains Neanderthal (Green et al. 2010) or Denisovan (Reich et al. 2010) ancestry as a direct result of admixture with these archaic species. Genes involved in immune response (Abi-Rached et al. 2011), pathogen defense (Enard and Petrov 2018), and protection from sun exposure (Dannemann and Kelso 2017) have introgressed from archaic humans as a result of these ancient hybridization events. One of the most striking conclusions from the many studies on human/Neanderthal or human/Denisovan admixture is that limited introgression can have significant genomic and phenotypic impacts.

Gene transfer across species boundaries has clearly taken place in *S. haematobium*. Although we see no evidence of early generation hybrids, we see a signature of ancient introgression from *S. bovis* in all Nigerien miracidia examined. We demonstrate that an introgressed M8 metalloprotease (invadolysin; Smp_127030) expressed in adult worms has spread to high frequencies in *S. haematobium* populations in Niger, and that parasites bearing introgressed *S. bovis*-derived alleles have a strong selective advantage over those carrying native *S. haematobium* alleles. Understanding the parasite phenotype conferred by the introgressed *S. bovis* alleles and the nature of selection driving the spread of these *S. bovis* alleles into *S. haematobium* populations is a high priority.

## Materials and Methods

### Ethics Statement

For the Niger sample collection, ethical clearance was obtained from the Niger National Ethical Committee, in combination with the St Mary’s Hospital Local Ethics Research Committee (part of the Imperial College London Research Ethics Committee [ICREC]; EC No. 03.36, R&D No. 03/SB/033E) in London, United Kingdom, in combination with the ongoing Schistosomiasis Control Initiative and Schistosomiasis Consortium for Operational Resaerch (SCORE) activities. For the Zanzibar sample collection, ethical approval was obtained from the Zanzibar Medical Research and Ethics Committee (ZAMREC, reference no. ZAMREC 0003/Sept/011) in Zanzibar, United Republic of Tanzania, the “Ethikkomission beider Basel” (EKBB, reference no. 236/11) in Basel, Switzerland, the Institutional Review Board of the University of Georgia (project no. 2012-10138-0), and registered at the International Standard Randomized Controlled Trial (Register Number ISRCTN48837681). Within both Niger and Zanzibar, all aspects of sample collections were carried out in the framework of the disease control activities implemented and approved by the local Ministry of Health and adopted by regional and local administrative and health authorities.

The study participants were informed about the study objectives and procedures. Written consent was obtained from parents prior to sample collection from children. Participation was voluntary and children could withdraw or be withdrawn from the study at any time without obligation. All children were offered PZQ (40 mg/kg single oral dose) treatment in the frame of the following school-based or community-wide treatment carried out by the Ministry of Health.

### Sample Collection

This study used archived miracidia samples from Niger and Zanzibar (Tanzania) fixed on Whatman FTA cards archived within the SCAN (Emery et al. 2012). From Zanzibar, encompassing both the Unguja and Pemba islands, the *S. haematobium* miracidia were collected as part of the SCORE population genetics studies in 2011 from 26 locations spaced up to 160.9 km apart. From Niger, the *S. haematobium* miracidia were collected in 2013 from school-aged children from 10 locations located up to 125 km apart. These samples were also collected as part of SCORE population genetic studies within a gaining and sustaining control of schistosomiasis project in Niger and also as part of monitoring and evaluation activities carried out by the Schistosomiasis Control Initiative. To capture maximum diversity we used a single miracidium from 96 individuals (*n*_Zanzibar_ = 48, *n*_Niger_ = 48). *Schistosoma haematobium* eggs were harvested from each infected urine sample by sedimentation or filtration, put into fresh water and then exposed to light to facilitate miracidial hatching. Miracidia were captured individually, under a binocular microscope, in 3 µl of water and spotted onto indicating Whatman FTA Classic Indicating cards (GE Healthcare Life Sciences, UK) using a micropipette, dried and archived in SCAN (Emery et al. 2012).

### Library Prep and Sequencing

We used whole-genome amplification of single miracidia dried on FTA cards followed by exome capture and sequencing (Illumina 2500) to generate genome-wide sequence data following methods described in Le Clec’h et al. (2018). The exome capture probe set (SureSelect design ID: S0742423) was designed using the published reference genome sequence for *S. haematobium* (SchHae_1.0, GCA_000699445.1, lab strain, originally from Egypt). The exome capture probe set included 156,004,120-bp RNA baits from the nuclear genome and 67 from the mitochondrial genome, covering 96% (62,106/64,642) of the exons and accounting for 94% of the exome length (15,002,706 bp/15,895,612 bp). Each captured exon was covered by an average of 2.59 120-bp baits (Le Clec’h et al. 2018).

In addition to exome capture, we generated whole-genome sequence data for twelve samples, six from each population (Niger and Zanzibar). Libraries were multiplexed and paired end 150-bp reads were sequenced on a single Illumina NextSeq flowcell. In addition to the whole-genome and exome sequence data generated above, we gathered available genome sequence data from the NCBI Short Read Archives for six other species of *Schistosoma* in the *haematobium* group, including *S. bovis* (ERR119622, ERR103048, and ERR539853), *S.* c*urassoni* (ERR310937 and ERR119623), *S. haematobium* (ERR084970, ERR037800, and SRR433865), *S. intercalatum* (ERR539854, ERR539856, and ERR119613), *S. guineensis* (ERR119612, ERR539850, and ERR539852), *S. mattheei* (ERR539851, ERR539855, ERR539857, and ERR103051), and *S. margrebowiei* (ERR310940) in 20 separate libraries (Young et al. 2012; Coghlan et al. 2019).

### Computational Environment

We conducted all analyses on a high-performance computing cluster within a Singularity container or Conda environment. Environmental recipe files, custom programs, and shell scripts are at https://github.com/nealplatt/sH_hybridization (v1.0; doi:10.5281/zenodo.2536390; last accessed 9 July 2019).

### Variant Discovery, Filtration, and Phasing

We trimmed sequence reads with Trimmomatic v0.36 (Bolger et al. 2014) so that the leading and trailing base calls had phred-scaled quality scores >10 and the phred score was >15 over a 4-nt sliding window. After trimming, we mapped paired and singleton reads to the reference *S. haematobium* genome (SchHae_1.0, GCA_000699445.1, lab strain, originally from Egypt). BWA v0.7.17-r1188 (Li and Durbin 2009) and merged into a single BAM file using SAMtools v1.7 (Li et al. 2009). GATK v4.0.1.1 (McKenna et al. 2010) was used to add read group information and mark duplicate reads from each library. Where possible, we added complete read group information based on information contained within the FASTQ header. In some cases (primarily the public data) pseudo read groups were created to differentiate each library.

We used GATK’s Haplotypecaller (McKenna et al. 2010) for variant discovery. Variant discovery was restricted to target regions of the *S. haematobium* assembly using the –L option. Target regions were identified by combining all adjacent, exome probe locations within 500 bases of one another into larger intervals with BEDtools v2.27.1 (Quinlan and Hall 2010). Each interval was genotyped using GATK’s GenotypeGVCFs and combined into a single VCF for filtering using GATK’s MergeVcfs. Low quality SNP genotypes were filtered using GATK’s VariantFiltration with the following filters: variant quality normalized by depth (QD < 2.0), strand bias (FS > 60.0), mapping quality (MQ < 40.0), mapping quality rank sum (MQRankSum < −12.5), and read position rank sum (ReadPosRankSum < −8.0). We used VCFtools v 0.1.15 (Danecek et al. 2011) to remove sites with high rates of missing data (>20%), multiallelic sites, and individuals with low call rates (>15% missing data). All indels were excluded from downstream analyses.

The published *S. haematobium* (SchHae_1.0) and *S. mansoni* (SM_V7) assemblies vary greatly in quality, despite a high degree of synteny between the two genomes (Young et al. 2012). We aligned the two genomes using progressiveCactus v0.0 (Paten et al. 2011) using default parameters to leverage the contiguity of the *S. mansoni* assembly. The HAL alignment file was used to lift SNP coordinates between the assemblies using progressiveCactus’ halLiftover module. We removed multiposition SNPs, those that align between assemblies in something other than a 1:1 relationship, from downstream analyses. We used LD decay curves to examine biases introduced during the coordinate liftover by comparing LD from SNPs associated with each assembly. SNPs mapping to the *S. mansoni* autosomes (chr1-7) were extracted using VCFtools v0.1.15. The square of the correlation coefficient (*r^2^*) was calculated for all SNPs within 1.5 Mb of each other for each data set using PLINK v1.90b4 (Purcell et al. 2007). The 1.5-Mb cutoff was chosen since it represented the largest scaffold in the *S. haematobium* assembly. After confirming concordance between the original and *S. mansoni*-lifted data sets, we used Beagle v4.1 (Browning and Browning 2016) to impute missing SNPs and phase haplotypes for each autosomal chromosome. Imputations and phasing occurred in sliding windows of 300 SNPs and a step size of 30 with 250 iterations per window.

### Mitotype Assignment

Unique filters were used to manage the mitochondrial SNPs. First, the mitochondrial contig was identified from the *S. haematobium* assembly using the *haematobium* versus *mansoni* whole-genome alignment and confirmed using NCBI’s BLAST server. Due to low genotyping rates within the Nigerien samples, mitochondrial SNPs were filtered so that the genotyping rate per site was reduced from 85% to 25%. Putative mitochondrial haplotypes (mitotypes) were generated by removing any heterozygous sites, if present. Given previously described rates of mitochondrial introgression and divergence between *S. bovis*, *S. curassoni* and *S. haematobium*, we mapped filtered reads directly to the *S. haematobium* mitochondrial contig (AMPZ01026399.1) and a previously generated *S. curassoni* mitochondria sequence (AP017708.1). Full-length *S. bovis* mitochondrial genomes are not currently available through NCBI GenBank (last accessed April 23, 2018). *Schistosoma bovis*/*curassoni* or *haematobium* mitotypes were classified using the ratio of reads mapping to each mitochondrial reference and confirmed by Sanger sequencing the mitochondrial, cytochrome C oxidase 1 (*cox1*) gene. *cox1* was amplified in a reaction containing 1× reaction buffer, 0.8 mM dNTPs, 1.5 mM MgCl2, 1 µM forward primer (Cox1_schist_5′; CTT TRG ATC ATA AGC G), and 1 µM reverse primer (Cox1_schist_3′; TAA TGC ATM GGA AAA AAA CA) and under the following reaction conditions: 2 min, 95 °C initial denaturation, 35 cycles of a 30 s, 95 °C denaturation, 30 s, 52 °C annealing phase, and a 1 min, 72 °C extension, followed by a final 7 min, 72 °C extension cycle (Lockyer et al. 2003). Amplification and fragment size were confirmed on a 1.5% agarose gel. Bidirectional sequences were generated for each fragment with the Cox1_schist_5′ and Cox1_schist_3′ primers by the Eurofins (Eurofins-MWG) sequencing service. All *cox1* sequences were compared with available sequences on GenBank for species identification.

### Population Structure, Admixture, and Ancestry Assignment

Summary statistics were calculated using filtered autosomal SNPs with minor allele frequency >5%. *F*_IS_ was calculated for each sample using VCFtools and the “–het” option. The scikit-allel v1.1.10 (Anopheles gambiae 1000 Genomes Consortium 2017) Python library was used to calculate *F*_ST_ between the Zanzibari and Nigerien populations of *S. haematobium*. Genome-wide values for *F*_ST_ were averaged from blocks of 100 variants and local calculations were generated from sliding windows of 250- and 50-kb steps. Relationships between samples were visualized in genotypic space with a PCA of unlinked SNPs. Linked sites within sliding windows of 25 SNPs and a pairwise *R*^2^ value >0.2 were filtered using PLINK v1.90b4 (Purcell et al. 2007). Since comparison between species can overwhelm population level clustering, separate PCAs were generated from previously published *Schistosoma* species (Young et al. 2012; Coghlan et al. 2019), and the exome data from the *S. haematobium* miracidia (Niger and Zanzibar) presented herein. Nucleotide diversity (*π*) was calculated per site and in sliding windows of 50 kb with 25-kb steps using VCFtools.

Levels of admixture were calculated using ADMIXTURE v1.3.0 (Alexander et al. 2009). The number of populations (*K*) was explored from 1 to 20 and cross-validation between 1,000 replicates was used to identify the most robust measure of *K*. The 3-population test *f*_3_ as implemented in the scikit-allel was used to identify admixture in the Nigerien and Zanzibar *S. haematobium* populations (Reich et al. 2009). *F*_3_, standard errors, and *Z*-scores were calculated from block-jackknife replicates of 100 SNPs. We used the scikit-allel package to calculate *D* statistics and compare possible introgression from *S. bovis* or S. *curassoni* into *S. haematobium*. Standard error and *Z*-scores were calculated by block-jackknife replicates in windows of 100 SNPs. In addition to genome-wide averaged, *D* was calculated in local, sliding windows of 50 SNPs with 25 SNP steps. Finally, PCAdmix v1.0 (Brisbin et al. 2012) was used for SNP ancestry assignment. For individuals in the admixed Nigerien population, ancestry was assigned in sliding windows across each chromosome to one of two parental populations defined by “pure” *S. haematobium* (represented here by samples from Zanzibar) and *S. bovis*. For validation, we included five, randomly selected, Zanzibari *S. haematobium* samples with the assumption that the entire genome should be assigned Zanzibari ancestry. Ancestry was assigned to windows of 30 SNPs at a time, with a minimum assignment threshold of 99%.

### Selection

We quantified selection across the *S*. *haematobium* exome using two independent methods. BayeScan v2.1 (Foll and Gaggiotti 2008) proposes two demographic models to explain allele frequency differences between at each locus. The first model assumes allele frequency differences are due to drift, whereas the other model adds an additional parameter (*α*) to account for selection. A Markov chain Monte Carlo was used to generate a posterior distribution of the alpha parameter such that significant deviations from zero indicate directional selection. We examined selection by comparing the Zanzibar and Niger *S. haematobium* populations using 1,000 separate BayeScan runs (chains) to guarantee convergence on posterior distributions alpha. For each chain we used 50 pilot runs of 10,000 generations to generate starting priors and burnin time of 50,000 generations. Prior odds for the neutral models (*α* = 0) were set at 10:1. To minimize autocorrelation, samples were thinned by 20 and 50,000 samples were taken. Convergence between chains was examined in R using the *coda* package v0.19-1. A Gelman-Rubin convergence diagnostic score <1.1 was defined as an acceptable level of convergence a priori. In addition to BayeScan, we identified regions under selection in both the *S. haematobium* populations using cross-population extended haplotype homozygosity (xpEHH; Sabeti et al. 2007) score as implemented in the *rehh* v2.0.2 R package (Gautier and Vitalis 2012). When possible, alleles were polarized against an outgroup (*S. margrebowiei*) to define the ancestral state. If sites were heterozygous or missing in *S. margrebowiei* they were excluded. Regions under selection were defined using a −log_10_(*P* value) = 3 an an |xpEHH| > 3. Adjacent loci ≤12.5 kb apart were combined into a single locus.

### Dating Genome-Wide Introgression

*Schistosoma bovis* introgression tracts were identified using Python scripts available at https://github.com/nealplatt/sH_hybridization (release v1.0; doi:10.5281/zenodo.2536390). Briefly, derived (autapomorphic) *S. bovis* and Zanzibari *S. haematobium* alleles were identified and used to annotate Nigerien *S. haematobium* alleles. Introgression blocks were identified by finding the largest stretch of *S. bovis* or pleisiomorphic alleles that were unbroken by a Zanzibari *S. haematobium* allele. With these tracts, we estimated the number of generations since admixture (Schumer et al. 2016):

$$G=\frac{1}{\mathrm{LP}},$$

where *G* is generations, *L* is the average length of introgression tracts in Morgans, and *P* is the proportion of the genome from the major parent.

We dated time since divergence of the Chr4: 20,023,951–20,047,325 locus in the Nigerien *S. haematobium* populations using startmrca (Smith et al. 2018). We used a uniform recombination rate of 3.4 × 10^−8^ and mutation rate of 8.1 × 10^−9^. We centered the analysis on Chr4: 20,033,013 and included 1 Mb of upstream and downstream sequence. Markov chain Monte Carlo chains were run for 50,000 generations and limiting proposals to 20 standard deviations. Ten independent chains were run using the parameters described above. Estimates of divergence time were taken by discarding the first 40,000 generations of each run then thinning the remainder to 1 sample per 10 generations.

### M8 Peptidase and Invadolysin Gene Family Evolution

Invadolysin (Smp_127030) is a member of the M8 peptidase family of proteins (Pfam ID: PF01457) from the Pfam database (v32.0; last accessed 28 October 2018; Finn et al. 2014). We downloaded amino acid sequences for all platyhelminths and several outgroup taxa including *Homo sapiens*, *Mus musculus*, *Monodelphis domestica*, *Gallus gallus*, *Anolis carolinesis*, *Danio rerio*, *Drosophila melanogaster*, *Aedes aegypti*, and *Caenorhabditis elegans*. Sequences missing the HExxH active site (Rawlings and Barrett 1995) were removed from downstream analysis. Amino acid sequences were aligned with MUSCLE v3.8.1551 (Edgar 2004), and sites with <75% coverage were removed. We used RAxML v 8.2.10 (Stamatakis 2014) to complete 100 searches for the optimal tree using the PROTGAMMAWAG substitution model. Gene duplication evens were inferred using Mega7 (Kumar et al. 2016) and the gene duplication wizard. Duplications were categorized based on their presence in lineages leading to *Schistosoma* paralogs.

### Invadolysin (Smp_127030) Structure

We used Phyre2 (Kelley et al. 2015) in “intensive mode” to model the Nigerien *S. haematobium* invadolysin (Smp_127030) protein structure. We used the whole-genome resequencing data to identify SNPs at the invadolysin (Smp_127030) locus since the exome probes only targeted 76.1% of the coding region (1,722 of 2,262 bp). Pockets in the protein structure were identified with fpocket2 (Le Guilloux et al. 2009) as implemented in PHYRE2 web portal. After modeling the invadolysin (Smp_127030) protein structure, we examined the placement of amino acid differences fixed between the Zanzibari and Nigerien *S. haematobium* populations with PyMol v2.2.3 (DeLano 2002).

### Invadolysin (Smp_127030) Expression

We quantified gene expression of Smp_127030 in *S. haematobium* using previously published data (Young et al. 2012) from schistosome eggs, and adult worms from both sexes (SRA accessions: SRX3632877, SRX3632879, and SRX3632881). Reads were mapped to the *S. haematobium* genome (SchHae_1.0) using hisat v2.1.0 (Kim et al. 2015). Transcripts were assembled and gene expression was normalized and quantified using Stringtie v1.3.4 (Pertea et al. 2015). Gene coordinates were lifted from the *S. mansoni* assembly to identify which of the Stringtie predicted transcripts were invadolysin (Smp_127030) using progressiveCactus’ halLiftover and the whole-genome alignment generated (described above).

Given the limited gene expression data available for *S. haematobium*, we examined expression of Smp_127030 and other invadolysin paralogs in *S. mansoni* (Berriman et al. 2009). We used RNA-seq data from miracidia (Wang et al. 2013), cercariae (Protasio et al. 2012), schistosomula (3 h, 24 h, in vitro transformation), sporocysts (48 h in vitro transformation), juveniles (single sex; 18, 21, 28 days old), and adults (single sex, mixed infections; 38 days) (Protasio et al. 2012; Wang et al. 2013; Protasio et al. 2017). We aligned the data using STAR v2.5.4b (Dobin et al. 2013). STAR references were prepared using the v7 *Schistosoma mansoni* genome the related v7.1 annotation (Coghlan et al. 2019) which was converted to the GTF format using gffread from cufflinks v2.2.1 (Trapnell et al. 2010) and either an overhang (-sjdbOverhang) of 75 (used for schistosomula and cercariae data) or 99 (used for all other libraries). We used RSEM (Li and Dewey 2011) to compute transcript per million counts for each isoform.

## Code Availability

Scripts, code, and environmental files are available at https://github.com/nealplatt/sH_hybridization (release v1.0; doi:10.5281/zenodo.2536390; last accessed 9 July 2019).

## Supplementary Material

Supplementary data are available at *Molecular Biology and Evolution* online.

## Supplementary Material

msz154_Supplementary_Data

## Notes

Sequence data are deposited in NCBI’s under BioProject accessions PRJNA508633 and PRJNA443709. Mitochondrial genomes are available in GenBank (MK253567–MK253578).
