# Supplementary material for: Ancient Hybridization and Adaptive Introgression of an Invadolysin Gene in Schistosome Parasites
Source: Mol Biol Evol. 2019 Jun 27;36(10):2127–42. doi: 10.1093/molbev/msz154 (PMC6759076; doi:10.1093/molbev/msz154)
Supplement: msz154_Supplementary_Data [file msz154_supplementary_data.zip › sh_exomics_SUPFIGS_2019-06-12.pdf]

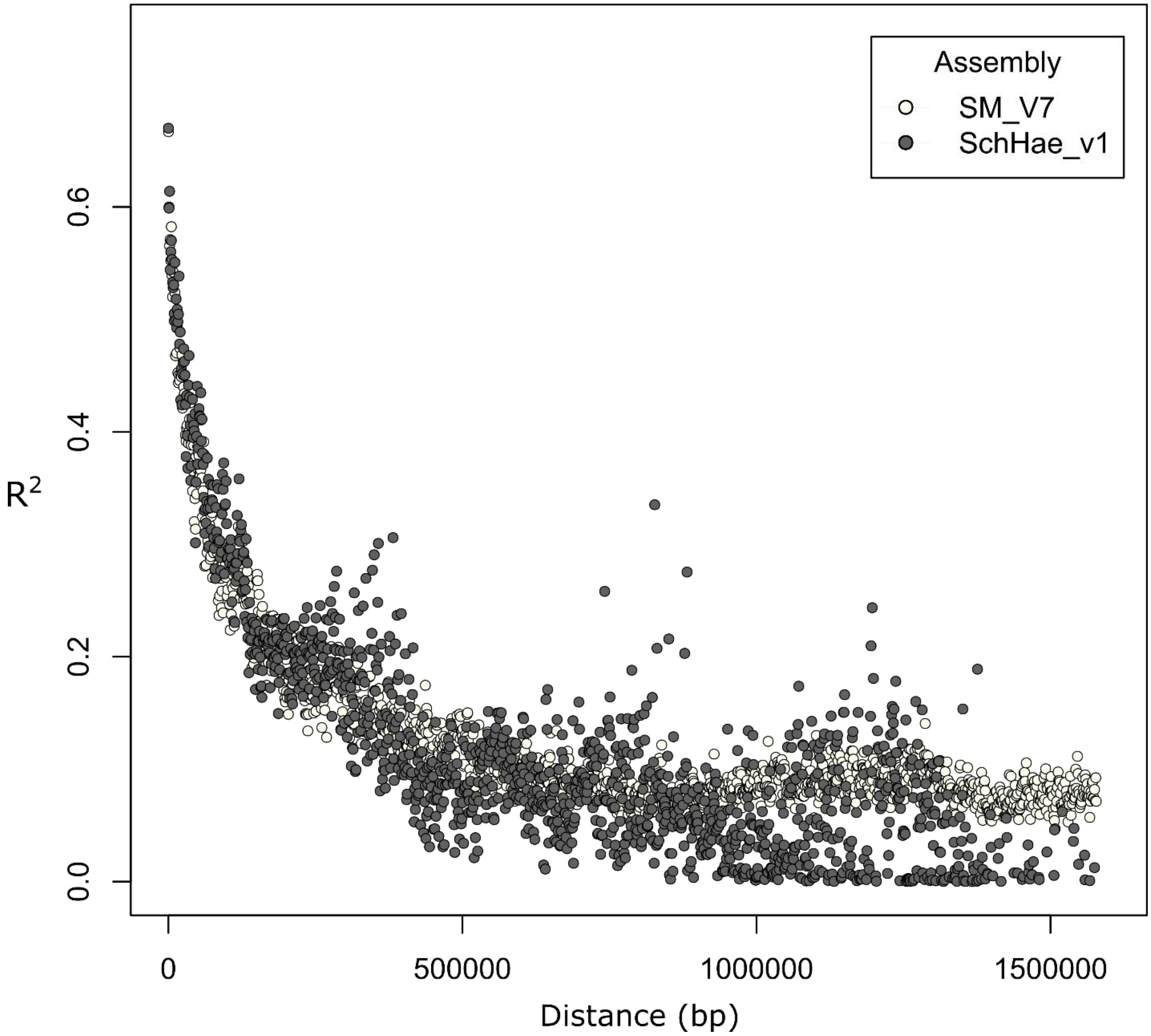

Supplemental Figure S1. Comparison of linkage disequilibrium (LD) between different genome assemblies. SNPs were genotyped using the current *Schistosoma haematobium* assembly (SchHae_v1). The *S. mansoni* genome (SM_V7) is a more contiguous assembly with most sequences contained in chromosomal length scaffolds. To take advantage of this contiguity, SNP coordinates were lifted from *S. haematobium* to the *S. mansoni* coordinates. LD was quantified from the *S. haematobium* SNPs on both assemblies. LD decays at similar rates for each set of coordinates, indicating a degree of synteny between the two genomes.

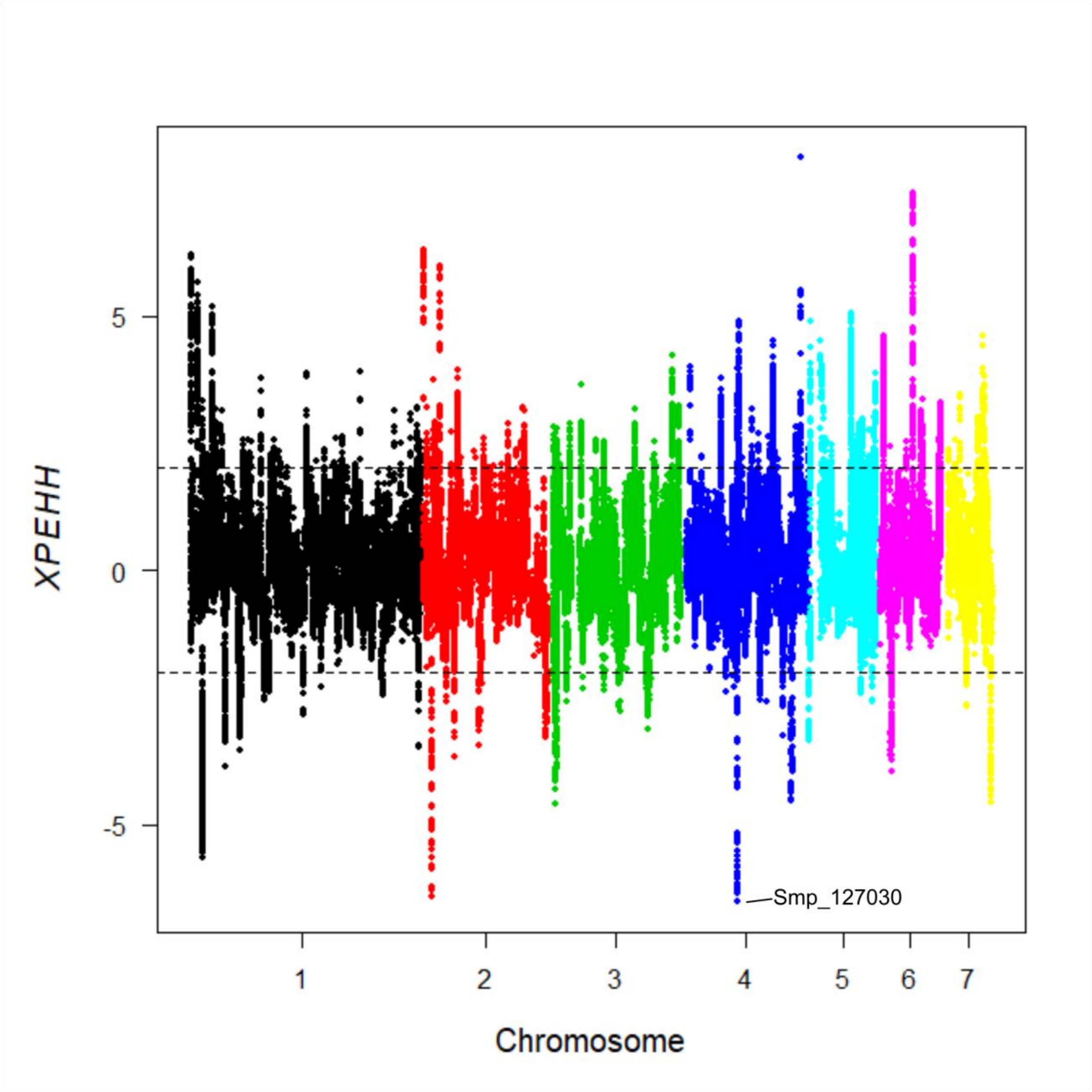

Supplemental Figure S2. Selection across the genome between Zanzibari and Nigerien *S. haematobium* populations. Selection across the genome was measured using cross population extended haplotype homozygosity (xpEHH). xpEHH > 2 indicates directional selection in the Zanzibari population and xpEHH < -2 indicates directional selection in the Nigerien *S. haematobium* population. Strong signals of selection are present throughout the genome, but the strongest signal of directional selection in the Nigerien population is on Chr4 and spans the invadolysin (Smp_127030) gene.

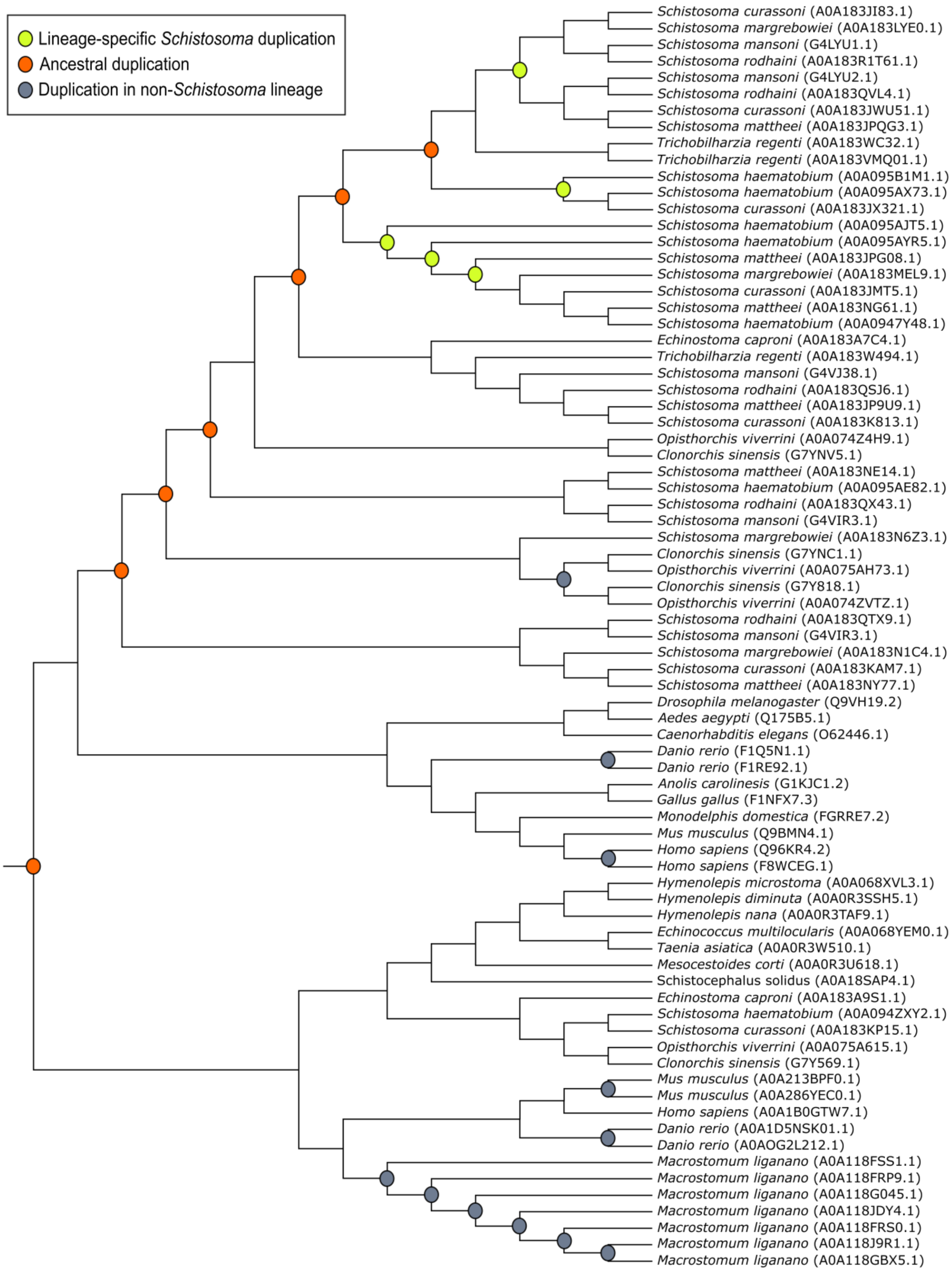
Lineage-specific Schistosoma duplication
Ancestral duplication
Duplication in non-Schistosoma lineage
Schistosoma curassoni (A0A183JI83.1)
Schistosoma margrebowiei (A0A183LYE0.1)
Schistosoma mansoni (G4LYU1.1)
Schistosoma rodhaini (A0A183R1T61.1)
Schistosoma mansoni (G4LYU2.1)
Schistosoma rodhaini (A0A183QVL4.1)
Schistosoma curassoni (A0A183JWU51.1)
Schistosoma mattheei (A0A183JPQG3.1)
Trichobilharzia regenti (A0A183WC32.1)
Trichobilharzia regenti (A0A183VMQ01.1)
Schistosoma haematobium (A0A095B1M1.1)
Schistosoma haematobium (A0A095AX73.1)
Schistosoma curassoni (A0A183JX321.1)
Schistosoma haematobium (A0A095AJT5.1)
Schistosoma haematobium (A0A095AYR5.1)
Schistosoma mattheei (A0A183JPG08.1)
Schistosoma margrebowiei (A0A183MEL9.1)
Schistosoma curassoni (A0A183JMT5.1)
Schistosoma mattheei (A0A183NG61.1)
Schistosoma haematobium (A0A0947Y48.1)
Echinostoma caproni (A0A183A7C4.1)
Trichobilharzia regenti (A0A183W494.1)
Schistosoma mansoni (G4VJ38.1)
Schistosoma rodhaini (A0A183QSJ6.1)
Schistosoma mattheei (A0A183JP9U9.1)
Schistosoma curassoni (A0A183K813.1)
Opisthorchis viverrini (A0A074Z4H9.1)
Clonorchis sinensis (G7YNV5.1)
Schistosoma mattheei (A0A183NE14.1)
Schistosoma haematobium (A0A095AE82.1)
Schistosoma rodhaini (A0A183QX43.1)
Schistosoma mansoni (G4VIR3.1)
Schistosoma margrebowiei (A0A183N6Z3.1)
Clonorchis sinensis (G7YNC1.1)
Opisthorchis viverrini (A0A075AH73.1)
Clonorchis sinensis (G7Y818.1)
Opisthorchis viverrini (A0A074ZVTZ.1)
Schistosoma rodhaini (A0A183QTX9.1)
Schistosoma mansoni (G4VIR3.1)
Schistosoma margrebowiei (A0A183N1C4.1)
Schistosoma curassoni (A0A183KAM7.1)
Schistosoma mattheei (A0A183NY77.1)
Drosophila melanogaster (Q9VH19.2)
Aedes aegypti (Q175B5.1)
Caenorhabditis elegans (O62446.1)
Danio rerio (F1Q5N1.1)
Danio rerio (F1RE92.1)
Anolis carolinesis (G1KJC1.2)
Gallus gallus (F1NFX7.3)
Monodelphis domestica (FGRRE7.2)
Mus musculus (Q9BMN4.1)
Homo sapiens (Q96KR4.2)
Homo sapiens (F8WCEG.1)
Hymenolepis microstoma (A0A068XVL3.1)
Hymenolepis diminuta (A0A0R3SSH5.1)
Hymenolepis nana (A0A0R3TAF9.1)
Echinococcus multilocularis (A0A068YEM0.1)
Taenia asiatica (A0A0R3W510.1)
Mesocestoides corti (A0A0R3U618.1)
Schistocephalus solidus (A0A18SAP4.1)
Echinostoma caproni (A0A183A9S1.1)
Schistosoma haematobium (A0A094ZXY2.1)
Schistosoma curassoni (A0A183KP15.1)
Opisthorchis viverrini (A0A075A615.1)
Clonorchis sinensis (G7Y569.1)
Mus musculus (A0A213BPF0.1)
Mus musculus (A0A286YEC0.1)
Homo sapiens (A0A1B0GTW7.1)
Danio rerio (A0A1D5NSK01.1)
Danio rerio (A0AOG2L212.1)
Macrostomum liganano (A0A118FSS1.1)
Macrostomum liganano (A0A118FRP9.1)
Macrostomum liganano (A0A118G045.1)
Macrostomum liganano (A0A118JDY4.1)
Macrostomum liganano (A0A118FRS0.1)
Macrostomum liganano (A0A118J9R1.1)
Macrostomum liganano (A0A118GBX5.1)

Supplemental Figure S3. A phylogenetic tree of M8 metalloproteases, including invadolysin (Smp_127030), in platyhelminths and selected outgroup species. Gene duplications are shown at each node with a circle and colored based on its presence in Schistosoma. Of the 77 sequences examined, paralogs in *S. curassoni*, *haematobium*, *mansoni*, *margrebowiei*, *mattheei*, and *rodhaini* accounted for almost half (n = 34). Duplications were classified based on the presence of schistosomes at the termini. Ancestral duplications are at nodes containing schistosomes and other taxa, lineage specific duplications are at nodes containing only schistosomes, and duplications at nodes without schistosomes. Multiple, lineage-specific gene duplications are necessary to explain the distribution of metalloproteases in *Schistosoma*.

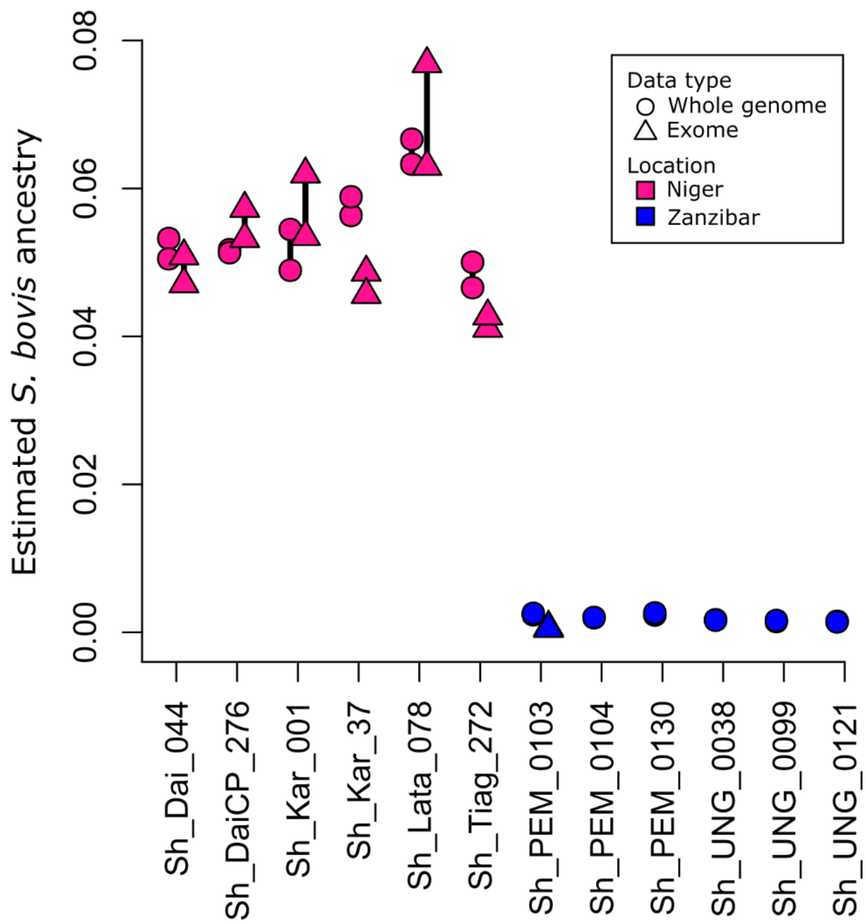

Supplemental Figure S4. Comparing *S. bovis* ancestry estimates between whole genome and exome data. Whole genome and exome data was generated for 12 *S. haematobium* individuals. To identify potential biases from either data type we compared the *S. bovis* ancestry estimates generated by PCAdmix. Ancestry estimates from exome data were available for all Nigerien and one Zanzibari *S. haematobium*. Ancestry estimates from exome data were not available for five Zanzibari *S. haematobium* since they were designated in the parental population. Ancestry estimates were consistent across both data types with the largest variation being 0.015 (1.5%).

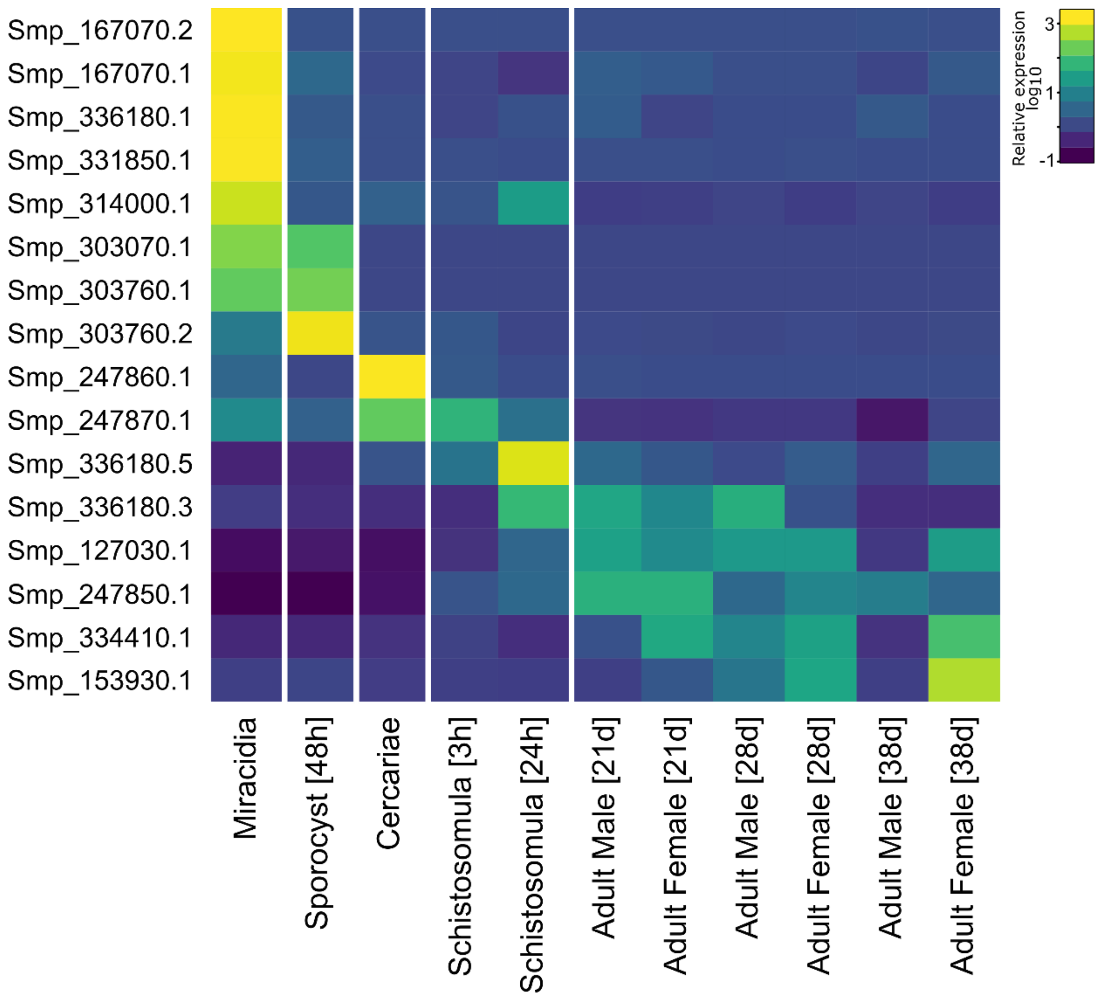

Supplemental Figure S5. Expression of invadolysin paralogs in *S. mansoni*. Relative expression values for each transcript are shown at major life stages. Different invadolysin paralogs appear to exhibit a continuum of stage specific expression. Invadolysin (Smp_127030) is primarily expressed in *S. mansoni* adult worms. "Smp" numbers refer to the accessions for the *Schistosoma mansoni* homologs.
